# Will Widgets and Semantic Tagging Change Computational Biology?

**DOI:** 10.1371/journal.pcbi.1000673

**Published:** 2010-02-26

**Authors:** Philip E. Bourne, Bojan Beran, Chunxiao Bi, Wolfgang Bluhm, Roland Dunbrack, Andreas Prlić, Greg Quinn, Peter Rose, Raship Shah, Wendy Tao, Brian Weitzner, Ben Yukich

**Affiliations:** 1 Skaggs School of Pharmacy & Pharmaceutical Sciences, University of California San Diego, La Jolla, California, United States of America; 2 San Diego Supercomputer Center, University of California San Diego, La Jolla, California, United States of America; 3 Department of Chemistry and Chemical Biology, Rutgers, State University of New Jersey, Piscataway, New Jersey, United States of America; 4 Institute for Cancer Research, Fox Chase Cancer Center, Philadelphia, Pennsylvania, United States of America; National Cancer Institute, United States of America and Tel Aviv University, Israel

We argue here, through the use of several examples from our work in support of structural biology, that the answer to the question posed by the title of this *Perspective* is a resounding yes. The discussion that follows is aimed primarily at those of the journal's readers who are biological resource developers and Web page developers interested in developing the richest possible Web pages. However, those of you who simply use biological resources might find this a helpful discussion in understanding what is on the horizon. Whatever your interest, please let us hear your opinion on the question posed by this *Perspective* through the associated comment feature.

We define a widget as a simple piece of code that can be embedded into a Web page or desktop to provide functionality that is derived from another Web site. To put widgets into perspective with other technologies, widgets share the portability and usability of an applet but are typically simpler. Similarly, widgets provide some of the functionality of products like Microsoft SharePoint, but are usually nonproprietary. Here a semantic tag is defined as a specific type of widget that brings some semantic information into the Web page or desktop from another Web site. Consider several simple examples (one desktop widget and the rest launched through a Web browser) we have developed recently that can be found (with others) on the RCSB Protein Data Bank (PDB) [1] Web site at http://www.pdb.org/pdb/static.do?p=widgets/widgetShowcase.jsp to illustrate the point.

**Figure 1:**
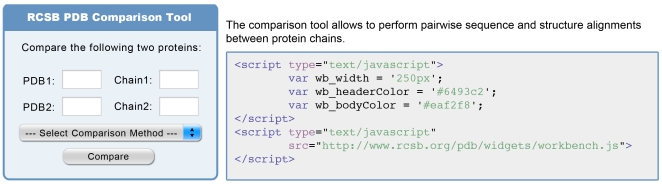
Sequence and structure comparison tool widget. The widget on the left is generated by the Javascript code on the right. Only the pointer (src) to the js file is required, the other lines allow the user to customize the look and feel of the widget to match their own Web page.

The first example widget was developed by the Protein Structure Initiative (PSI) Knowledgebase (KB) project (http://kb.psi-structuralgenomics.org/) [2]. The KB widget automatically detects new articles, structures, and features when the site updates on the third Thursday of each month. Embedded in a Web page it provides immediate access to the new features at the KB from any Web page. The second example widget is a dashboard widget contributed by Brian Weitzner and Roland Dunbrack (http://dunbrack.fccc.edu/dashpdb/). The Mac-OSX dashboard widget provides a simple way of querying the RCSB PDB or downloading a specific structure from the Macintosh dashboard application and as such represents a useful shortcut. The third example is illustrated in more detail in Figure 1.

As a user, this simple piece of code embedded in the Web page you are browsing enables you to run a complex application from another Web site, without leaving your current location. In this case it involves either comparing two protein sequences or two protein structures using a variety of methods supported by the remote Web site, in this case the RCSB PDB. Why is this compelling? In part because you do not need to undertake any work yourself. The remote provider is maintaining the applications, keeping the data current, and providing the widget, yet the application is available right there on your Web page. Most importantly it eradicates multiple versions of obsolete software. This is an application developer's nightmare as you try and support multiple obsolete versions of software from people who, for whatever reason, do not upgrade to the current version. The most current version is always there available to you on your Web page. You could just visit that Web site and do the same thing, so why the big deal? First, it brings the application to you; it is an example of drop technology (simply drop the application into your Web page) and it facilitates use. You do not have to remember where to go and possibly be faced by a series of complex choices—the widget can offer a simplified interface to a subset of features. Second, and more importantly, assuming the use of widgets takes off, you can customize your own Web page to take advantage of work done by a variety of other scientists each producing widgets. So for example, you could aggregate a variety of remote methods that perform sequence and structure comparison using a variety of widgets from a variety of reputable sources, thereby creating a single point of reference. Taking this a step further, you can create and customize workflows composed of different widgets in a plug and play environment. There is nothing fundamentally new in what we present here; widgets have been around since the early days of the Web. A display counting Web page hits is one example that has been used widely. What is new is the simplicity and hence ease of development and use of widgets and the general acceptance of this technology by the broader Internet community. There is still a small barrier for inclusion of widgets since it requires some knowledge of the Cascading Style Sheets (CSS) and/or Javascript. Even that can be overcome, witness iGoogle (http://www.google.com/ig), which is a good example of how this can work with a user customizing their home page from a variety of widgets (Google call them gadgets). Similarly, Facebook has drop-in widgets they call applications. As this is written, there is relatively little use of this technology by the computational biology community. We take that to mean that this is a new development, the full significance of which is yet to be appreciated (hence this article). Please beg to agree or differ by adding a comment.

Assuming widgets are a compelling development for the field, what is the potential and why would people use them? Addressing the latter question, Web resource developers are driven by getting eyeballs on their site; good Web statistics are a prerequisite for grant renewals. Although with widgets the eyeballs may no longer be directed at the site, but at a small component of the site integrated remotely. Nevertheless, their work is potentially made accessible to a larger number of users than would otherwise be the case and it registers Web hits on their own site. Addressing the former question, the potential, in our opinion, is to provide the opportunity to make some order out of the chaos that exists today. Users of computational biology resources face a bewildering array of resources, different interfaces, and a lot of features they will likely never use. Google obviously know how to do it best as proven by their search engine interface. But such simplicity applied to complex biological applications is not always possible, although extraction of suitable subsets of functionality into widgets may be possible. Further, and a dream perhaps, a few standards for widget development both on the server side and the presentation side could provide a productivity gain for the life sciences community who are increasingly dependent on these computational resources. Being optimistic, it might even bring to light in new ways the most authoritative and dependable resources. Being very optimistic we might see an end to the lack of persistence in computational biology resources that has been discussed previously [3]. Resources would use persistent URLs (PURLs) and their Application Program Interface (APIs) would conform to agreed upon standards.

**Figure 2:**
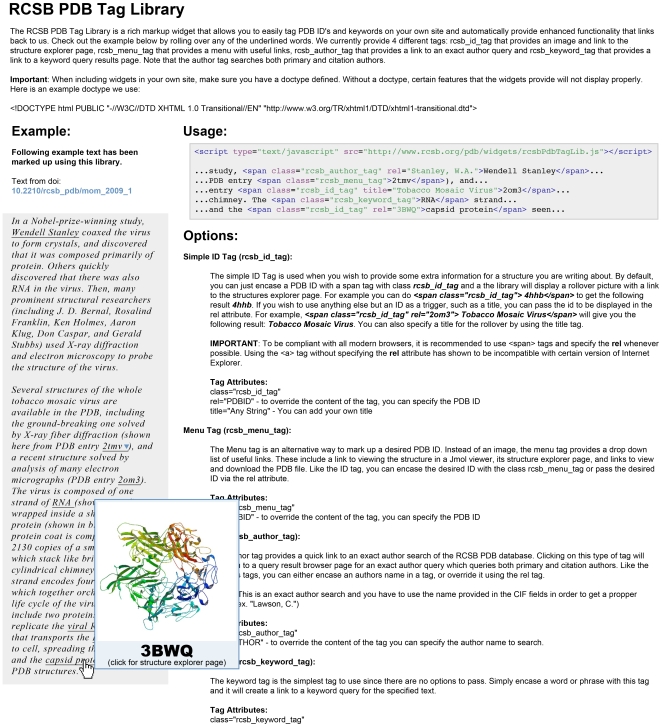
Semantic tagging. The box labeled usage shows how these tags are used in the html document. On the left is such a Web page (in italics) that has been semantically tagged. The Web page was created by David Goodsell as part of the RCSB PDB “Molecule of the Month” feature.

Talk is cheap; consider the specific example of semantic tagging, which we believe makes the argument for widgets even more compelling (Figure 2). Again here is an example from the RCSB Protein Data Bank.

There are four tags used in the document that illustrate how new life and comprehension can be bought to Web pages. This is done without any loss of context. As the author mouses over the Web page itself new information is bought forth from other resources defined by the Web page author. The author tag tied to *Stanley, W.A*. will return all entries in the PDB database that have been authored by the same person. The menu tag tied to entry *2tmv*, a specific structure in the PDB, brings up a menu of options associated with access to that entry, for example display the sequence of the entry, display a summary page from the PDB describing the structure, and so on. The keyword tag attached in this case to *RNA* will search for all instances of that keyword in the PDB and return associated structures. Finally the rcsb_id tag attached here to the term *capsid protein* will provide a thumbnail view of the molecule that can be selected for a more detailed view from the RCSB PDB. The intent here is to illustrate that a simple text document can be enriched to benefit the reader. Certainly such tags can be ignored, but they can also provide additional insights into the work described in the document through direct and contextual information from elsewhere, which in this case just happens to be a database of protein structures. If hyperlinks are considered powerful and the core of what makes the World Wide Web, this type of semantic tagging adds a new dimension to the Web. If semantic tagging were to take off, issues of name space might appear, but for now having exemplars that illustrate the power of the medium would seem an excellent first step. Imagine the day when such tags are added to research articles as they are written and are carried through to the final published paper. Perhaps the promise of the semantic Web will be realized. Time will tell, for now it would be good to see more computational biologists embrace and promote this technological development. What do you think?
